# Modeling and Analysis of a Novel Ultrasensitive Differential Resonant Graphene Micro-Accelerometer with Wide Measurement Range

**DOI:** 10.3390/s18072266

**Published:** 2018-07-13

**Authors:** Fu-Tao Shi, Shang-Chun Fan, Cheng Li, Xiao-Bin Peng

**Affiliations:** School of Instrumentation Science and Opto-electronics Engineering, Beihang University, Beijing 100191, China; 12171038@buaa.edu.cn

**Keywords:** graphene resonator, accelerometer, simulation analysis, MEMS/NEMS (micro/nanoelectromechanical systems) sensors

## Abstract

A novel, ultrahigh-sensitivity wide-range resonant micro-accelerometer using two differential double-clamped monolayer graphene beams is designed and investigated by steady-state simulation via COMSOL Multiphysics software in this paper. Along with stiffness-enhanced optimized folded support beams, two symmetrical 3-GPa prestressed graphene nano-beams serve as resonant sensitive elements with a size of 10 μm × 1 μm (length × width) to increase the acceleration sensitivity while extending the measurement range. The simulation results show that the accelerometer with cascade-connected graphene and proof-mass assembly exhibits the ultrahigh sensitivity of 21,224 Hz/g and quality factor of 9773 in the range of 0–1000 g. This is remarkably superior to previously reported studies characterized by attaching proof mass to the graphene components directly. The proposed accelerometer shows great potential as an alternative to quartz and silicon-based resonant sensors in high-impact and highly sensitive inertial measurement applications.

## 1. Introduction

Due to their direct frequency signal output, stable performance, excellent repeatability and high sensitivity, resonant accelerometers as an important development of inertial area have been widely applied in aviation, aerospace and automation fields. The resonance frequency-dependent resonant sensitive element is a key part of resonant accelerometers. At present, resonant sensitive elements are mainly composed of precision transverse elastic alloy, monocrystalline silicon, fused quartz and piezoelectric quartz crystal. Resonant accelerometers using these materials generally demonstrate low sensitivity and large sizes [1,2,3]. Therefore, in order to achieve miniaturization, wider acceleration range and better measurement accuracy, it is desirable to use novel materials possessing ultrathin thickness and outstanding electromechanical characteristics as the sensitive elements of resonant MEMS/NEMS accelerometers.

Graphene is a two-dimensional material with a single-layer thickness of 0.335 nm, which is the thinnest material known up to now [4]. Since monolayer graphene was first exploited in 2004 [5], graphene has been widely used because of its excellent mechanical [6], electrical [7], optical [8] and thermal properties [9]. In addition, graphene’s Young modulus is measured to be about 1.1 TPa, so that it can be stretched by 20% without breaking [10]. Thus, graphene provides possibilities to make high performance resonant sensors [11]. For example, Bunch et al. studied the mechanical properties of suspended single-layer graphene sheet over trenches, such as the natural frequency and the quality factor (*Q*), and then compared the results with the theoretical model of a vibrating beam under tension which indicated the feasibility of resonant graphene sensors [12]. Chen et al. investigated the mechanical vibration of a monolayer nano-mechanical resonator via electrical detecting [13]. Oshidari et al. also reported a graphene resonator that attached SU-8 proof mass to graphene and studied resonance frequency and quality factor variations changing with temperature [14]. Recently, Kown et al. used molecular dynamics method to analyze the mechanical response of graphene harmonic oscillator under nano-indentation. The simulation showed that the graphene resonator could be tuned by nano-pressure and initial displacement to a large extent [15,16]. Additionally, regarding the advantages of optical fiber measurement, Li et al. developed a fiber-tip Fabry–Perot resonator with multi-layer graphene diaphragm and analyzed the effect of structural parameters on the resonant characteristics [17].

Due to excellent resonant characteristics, graphene resonators have been explored to measure the acceleration. Kang et al. investigated highly sensitive resonant accelerometers based on graphene nanoribbon via classical molecular dynamics simulation. They pointed out that the acceleration was closely related with structural parameters and built-in strain of graphene nanoribbon [18]; however, the measured acceleration was at 10^15^ g level, which is improper for practical applications. Byun et al. also attached micro mass (4.0152 × 10^−22^–4.0152 × 10^−20^ g) to graphene nanoribbon to sense acceleration and examined the linear relationship between acceleration and resonance frequency on a log–log scale [19]. Unfortunately, the attached mass is too small to control and easily introduces impurities, which will negatively affect the vibration mode and responsive repeatability of graphene nanoribbon. After that, Lee et al. attached SU-8 proof mass to clamped graphene drum to evaluate the acceleration sensing [20]. Hurst et al. presented an SU-8 clamped graphene NEMS accelerometer which demonstrated a repeatable response to an input acceleration level of 1000–3000 g [21]. Note that, since larger acceleration measurement range will result in lower sensitivity, the graphene’s charge-dependent conductance is needed to amplify the capacitive current modulation. Hence, Kang et al. examined the dynamic characteristics of a designed crossroad-type resonant graphene accelerometer by using classical molecular dynamics simulations, which shows that resonance frequency will increase with the acceleration change at accelerations greater than 0.1 nm/ps^2^ [22]. However, it is difficult for this type of accelerometer to operate in process. Recently, Jie et al. attached rectangle-shape gold proof mass to a graphene sheet in micro level and studied the effect of the position and size of gold proof mass on the graphene resonant characteristics [23]. Nevertheless, the studies related with resonant graphene accelerometer are still relatively rare and primarily focus on acceleration-dependent resonant characteristics instead of integrated structure design. It should be noted that resonant graphene accelerometer studies reported previously applied proof mass to the graphene nanoribbon or graphene sheet directly to sense acceleration, which would limit the stability of accelerometer while vibrating and then easily damage the graphene sensitive element, thereby resulting in lower measurement accuracy.

Therefore, in this paper, we propose an architecture of differential resonant accelerometer by sensing graphene beams’ stretching applied by a proof mass. The COMSOL Multiphysics steady-state simulation of the proposal accelerometer without proof mass attached on graphene exhibits the acceleration sensitivity of 21,224 Hz/g and *Q* of 9773 in the defined range of 0–1000 g, which is significantly higher than the previously reported research where *Q* values were confirmed as 1000 [24], 3000 [25], 7000 [14] and 9000 [26], respectively.

## 2. Modeling the Designed Accelerometer

### 2.1. Design of Resonant Graphene Accelerometer

**Figure 1:**
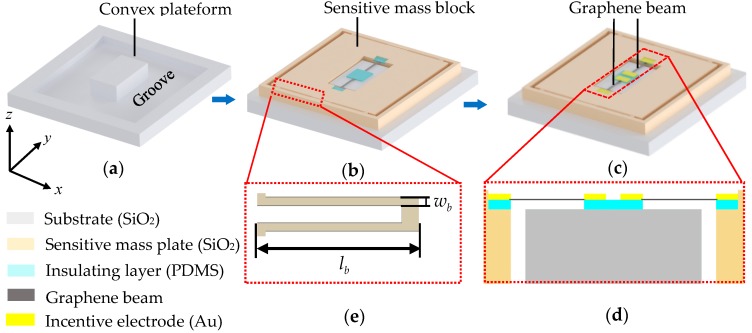
The model of a differential resonant graphene accelerometer. (**a**) The substrate of accelerometer; (**b**) etching sensitive mass plate and preparing insulating layers and graphene beams; (**c**) fabricating the integrated accelerometer; (**d**) enlarged *y*–*z* cross-sectional view of the central area of accelerometer; (**e**) partial enlarged drawing of one side folded support beam in inset.

Figure 1 shows the model of the differential resonant graphene accelerometer, wherein the presented accelerometer consists of a substrate, two double-clamped graphene beams (DCGBs), two pairs of incentive electrode pairs and a sensitive mass plate with a sensitive mass block and four folded support beams, as well as insulating layers. As shown in Figure 1a, a groove is etched in the substrate to eliminate the friction between substrate and sensitive mass plate under working condition, so as to form a rectangular convex platform on the substrate covered by the insulating layer. Note that the sensitive mass plate can be fixed to the substrate by using SIMOX (Separation by Ion Implantation of Oxygen) wafer bonding technology. The inside of the sensitive mass plate’s frame is then flush with the outside of the substrate’s groove, thereby making the center of the sensitive mass plate concentric with the center of substrate and ensuring the sensitive mass block is symmetrical along the center of accelerometer. The following process is to etch the sensitive mass plate in order to form a sensitive mass block, which is arranged to distribute symmetrically along the axial direction (*y*-axis) of accelerometer, and two grooves for placing graphene resonant beams. As shown in Figure 1c,d, the insulating layers are arranged not only in the central area of the substrate’s convex, but also in the grooves of the sensitive mass plate, which contributes to clamping the two ends of each graphene beam with corresponding incentive electrodes in the *y*-axis direction. In other words, the fabricated graphene resonant beams operate under double-clamped boundary condition. In this case, the substrate and sensitive mass plate could be fabricated by thermal growth oxidation [27,28], and the two DCGBs could be formed by chemical vapor deposition or thermal deoxygenation growth. Note that the incentive electrode pairs for stimulating the beam to resonant condition are placed on the ends of graphene resonant beams. In order to obtain high linearity, the support beams should have high axial stiffness and low cross sensitivity. Hence, as illustrated in Figure 1e, the folded support beam is adopted as the transmission structure of concentrated force caused by acceleration [29].

In particular, the designed resonant sensing structure is symmetrical along the center of accelerometer, thereby making the whole sensitive structure highly symmetrical. Combined with high axial stiffness and low cross-sensitivity of folded support beams, the sensing structure can fundamentally eliminate the sensitive mass block’s movement caused by the acceleration in other directions and the rotation around other sensitive axes. More importantly, it can guarantee not only high acceleration induced displacement sensitivity and smaller off-axis crosstalk, but also high resonance frequency. Furthermore, the differential resonant structure can enhance the signal intensity and improve nonlinear characteristics of graphene accelerometer, which contributes to increasing the sensitivity and measurement accuracy, as well as better robustness to the interference resulting from conjugate suppression and compensation effect.

### 2.2. Working Principle

Referring to the model in Figure 1, the substrate and sensitive mass plate form the main body of the accelerometer. The two driven DCGBs are placed along the axial direction (*y*-axis) of the sensitive mass plate in vacuum environment. The measured acceleration imposed on sensitive mass plate is converted to a concentrated force by the folded support beams in the sensitive mass plate as shown in Figure 1e. The concentrated force can make the sensitive mass block generate a small displacement, which could be described by the following equation

(1) $$x=\frac{F(a)}{K}=\frac{m\cdot a}{K}$$

where x is the displacement of the sensitive mass plate; F(a) is the concentrated force caused by acceleration; K is the equivalent stiffness of folded support beam; m is the mass of sensitive mass block; a is the measured acceleration. The stiffness of folded support beams is the key parameter determining the displacement (x), which can be determined as [30]

(2) $$K=2Eh\cdot {\left(\frac{w_{b}}{l_{b}}\right)}^{3}$$

where E is Young’s modulus of folded support beams; h is the beam thickness; $w_{b}$ is the beam width; $l_{b}$ is the length of unilateral beam. Since the whole length of the dual-beam structure ($L_{b}$) is $2l_{b}$, the corresponding total stiffness can be expressed as

(3) $$K_{total}=\frac{Eh{w_{b}}^{3}}{4{l_{b}}^{3}}$$

The displacement caused by the folded support beams will result in a movement in one end of the DCGB and then lead to axial stress changes in the graphene resonant beams. The resonant frequency ($f_{R}$) of DCGB will vary with the stress change in beams. Hence $f_{R}$ under initial stress ($F_{0}$) and concentrated force F(a) can be described as [18]

(4) $$f_{R}=\frac{n}{2L}\sqrt{\frac{F_{0}+F(a)}{\rho w}}=\frac{n}{2L}\sqrt{\frac{F_{0}+ma}{\rho w}}$$

where n is harmonic order; L is the length of DCGB; ρ is the mass density of graphene; w is the width of DCGB. In this way, when a certain axial acceleration causes axial push and pull loads imposed on the DCGB, the resonance frequency of DCGB will change accordingly, thereby resulting in a periodic electrical signal output through incentive electrodes electrically connected with graphene resonant beams. In this case, the applied acceleration can be derived by the measured resonance frequency of the DCGB via electrical detecting, along with Equation (4).

## 3. Structural Optimization and Simulation

The model is imported to COMSOL Multiphysics by utilizing “Livelink for Inventor” module, wherein the initial values of structural parameters of designed accelerometer are listed in Table 1. Each end of the DCGB is clamped by insulating layers and incentive electrode pairs in the axial direction (*y*-axis) of accelerometer. The end of the DCGB connected to the sensitive mass block is free and the other end is clamped. Assuming the density of graphene (ρ), Young’s modulus (E) and Poisson’s ratio (*v*) are 2208 kg/m^3^, 1.1 TPa and 0.41, respectively. In this case, we apply a concentrated force load to the sensitive mass block in axial direction (*y*-axis) to simulate the concentrated force induced by acceleration.

**Figure 2:**
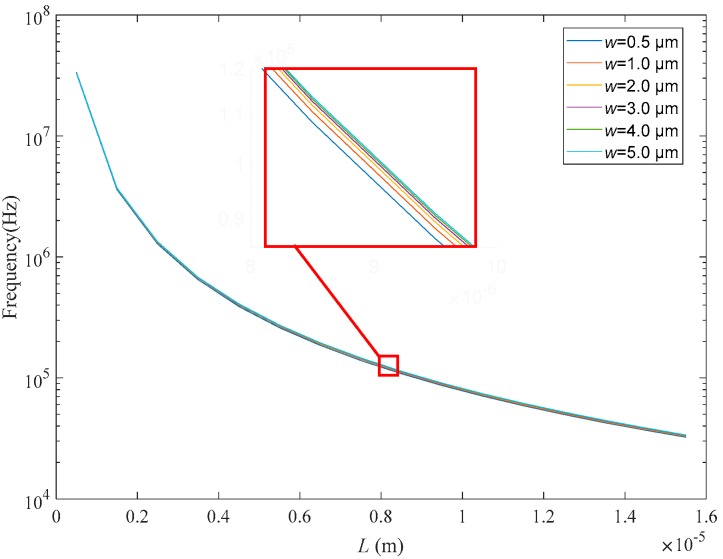
Natural frequency verse the length and width of graphene beam.

Graphene beam is a key element of resonant accelerometer. In view of the aspect ratio of graphene, the module “Membrane Interface” is selected in COMOSL, which is exclusively oriented to prestressed-ultrathin membrane simulation. Then, the module “Swept Mesh” is established by regulating the number, size and distribution of mesh in the thickness direction of membrane, thereby ensuring the computing integrity and accuracy of simulation. It is worth noting that, currently the COMSOL simulations have been widely adopted to investigate mechanical, optical and thermal behaviors of graphene-based beam, coating and ribbon with single-layer, few-layer or multi-layer thickness [31,32,33]. For these reasons, we can conclude that the resonant characteristics of the proposed graphene accelerometer obtained by COMSOL multiphysics simulation are effective and convincing in the manuscript. For achieving high resonance frequency, it is necessary to optimize the size of the graphene beam. In this paper, with regard to the graphene resonant beam with a monolayer thickness of 0.335 nm, we sweep its length (L) from 0.5 μm to 15 μm and width (w) from 0.5 μm to 5 μm. The frequency at first order mode is chosen to study the size effect on natural frequency of graphene beam. As illustrated in Figure 2, the natural frequency of graphene beam decreases with the beam length. Moreover, the frequency decreases dramatically when the length is shorter than 8 μm. In terms of width, there is no apparent difference in resonance frequency when the width varies from 0.5 μm to 5 μm. According to Jie et al. the free edges of graphene beam play a more important role than the clamped edges [23], so the beam width will have a weak impact upon the frequency. Consequently, in order to further estimate the structural characteristics of the beam for achieving observable vibration mode, we set the dimension of the beam as 10 μm × 1 μm × 0.335 nm (length × width × thickness), which contributes to the miniaturization of the accelerometer at this level.

**Figure 3:**
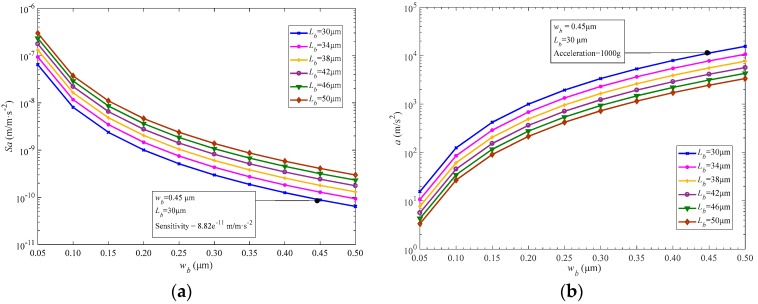
(**a**) Sensitivity and (**b**) acceleration as function of the dimension of support beam.

According to Equations (1) and (2), the dimension of folded support beam has an influence on the equivalent stiffness (K) and sensitivity ($S_{a}=x/a$). Furthermore, if the stiffness is too large, it would extend the acceleration measurement range, but apparently limit the sensitivity. On the contrary, the sensitivity will increase if the stiffness is small enough. For this reason, it is essential to optimize the dimension of the support beam. Note that, if the length of the folded beam is too short, such as lower than 30 μm, the folded beam cannot easily occur to compress because of large bending stiffness induced by a small aspect ratio. Moreover, the sensitive mass block tends to be suppressed to rotate along the sensing y-axis. In addition, it is also difficult to etch the folded beam whose width is thinner than 0.05 μm. Thus, we swept $L_{b}$ from 30 μm to 50 μm and $w_{b}$ from 0.05 μm to 0.5 μm, and then the sensitivity ($S_{a}$) and acceleration variation as a function of length ($L_{b}$) and width ($w_{b}$) were analyzed, as shown in Figure 3. Referring to Figure 3a, the sensitivity of the support beam decreases with the width, but increases with the length. In contrast, the measured acceleration in Figure 3b increases with the width, while decreasing with the length. In particular, the acceleration will increase by three orders of magnitude when the width increases from 0.05 μm to 0.5 μm, which would significantly restrict the measurement range. An ideal accelerometer should have higher acceleration sensitivity so that it can produce large displacement to acquire high resonance frequency. However, a larger sensitivity will limit the acceleration range. In consideration of previously reported studies [19,20,21], the measured largest acceleration available was up to 1000 g. As shown in Figure 3, when $w_{b}$ is wider than 0.45 μm and $L_{b}$ is shorter than 30 μm, the acceleration can be larger than 1000 g. However, both wider width and shorter length will reduce the sensitivity, as shown in Figure 3a. For these reasons, in order to achieve high sensitivity and wide acceleration range approaching 1000 g, the two fixed parameters ($w_{b}$ and $L_{b}$) are confirmed as 0.45 μm and 30 μm, respectively, for the subsequent characteristics simulation on resonant responses of the proposed accelerometer.

## 4. Performance Analysis and Discussion

### 4.1. Effect of Prestress

**Figure 4:**
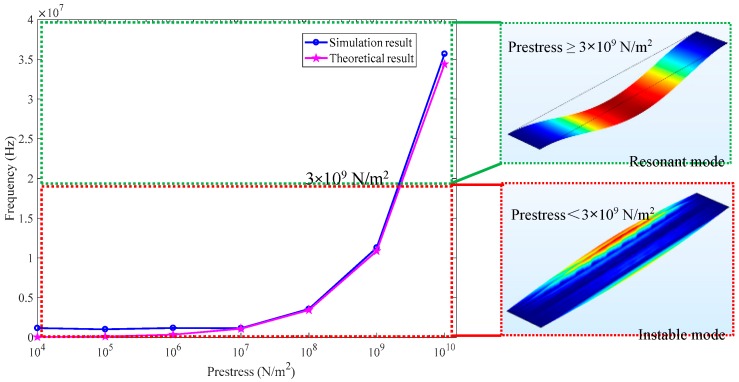
Prestress-induced resonance frequency change of graphene beam.

Given the resonant frequency shift closely depends on the prestress of graphene beam, a proper prestress should be determined. We investigated the frequency variations with the prestress ranging from 10^4^ N/m^2^ to 10^10^ N/m^2^ by simulation and theoretical model. It can be observed from Figure 4 that when the prestress is smaller than 10^7^ N/m^2^, there is no obvious frequency change. In contrast, when the prestress is larger than 10^7^ N/m^2^, the frequency will increase significantly with the prestress. Furthermore, according to Chen et al. [13], the two-dimensional mass density *ρ* of graphene represents the sum of the contributions from the graphene itself and any adsorbates. In this case, Equation (4) for DCGB with zero bending stiffness is solved by using a density value *ρ* (*ρ* = 2208.96 kg/m^3^) larger than theoretical value *ρ_graphene_* (*ρ_graphene_* = 2208 kg/m^3^) in this paper. Therefore, there is a small deviation existing between the simulation and theoretical results. That is, the former is slightly higher than the latter, as shown in Figure 4. Considering that the actual prestress of graphene will not reach 10^10^ N/m^2^ [7], the prestress should be determined in the range of 10^7^ N/m^2^ to 10^10^ N/m^2^. It should be mentioned that, as depicted in inset in Figure 4, the compressing DCGB has instable vibration mode when the prestress is smaller than 3 × 10^9^ N/m^2^ (3 GPa). For ensuring the DCGB under compression to work in resonant status with high natural frequency (1.954 × 10^7^ Hz) for the proposed accelerometer, the prestress of the designed graphene beam is confirmed as 3 × 10^9^ N/m^2^ (3 GPa). The prestress of DCGB could be applied by improving the surface roughness of insulating layers and electrodes [13,18] or employing hard-baking technology to induce strain on the DCGB by making the clamping blocks shrink during the hard-bake [20], which also contributes to enhancing the van der Waals attraction between the DCGB and the clamping blocks.

### 4.2. Performance and Analyses

**Figure 5:**
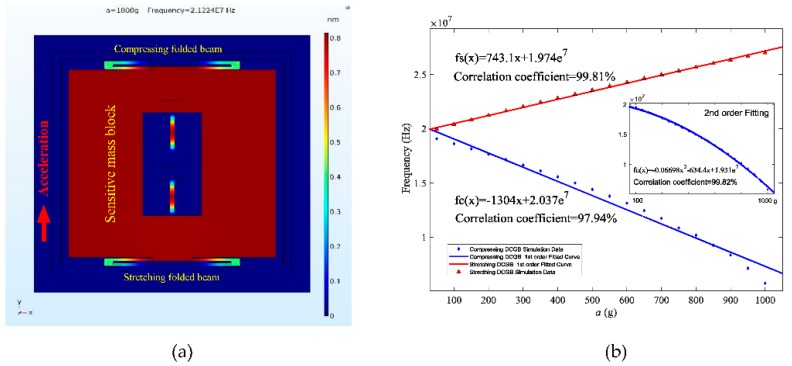
Simulation results of resonant accelerometer. (**a**) The displacement of sensitive proof mass of accelerometer under 1000 g acceleration; (**b**) resonance frequency variations of compressing and stretching DCGB as function of acceleration.

By utilizing the parameters as defined above, we further study the input-output performance of resonant accelerometer in response to measured acceleration. As shown in aforementioned Figure 5a, under the effect of the concentrated force caused by applied acceleration (1000 g), one folded support beam in the upper compresses and the other support beam stretches in axial direction (*y*-axis). The sensitive mass block in the center is driven into motion and causes the deformation of two support beams and two DCGBs, resulting in a distinct graphene beams’ resonance frequency change of 0.75 × 10^7^ Hz compared to natural frequency (1.95 × 10^7^ Hz).

Based on the established simulation model, we can achieve the resonance frequency variation of two DCGB's as a function of acceleration. From Figure 5b, the stretching and compressing DCGB have identical fundamental frequencies at zero acceleration. As the acceleration increases from 0 to 1000 g, the stretching DCGB’s resonance frequency increases linearly with the increase of the acceleration, while the compressing DCGB shows an inconspicuous nonlinear decline. Typically, graphene accelerometers will exhibit nonlinear vibrations in the presence of large strain, and the nonlinearity will increase with applied larger acceleration values [30,34,35]. In this case, the curve of compressing DCGB is fitted by a 1st order polynomial with a correlation coefficient of 97.94%. Then according to Equation (4), the frequency-acceleration response is further fitted by a 2nd order polynomial with a correlation coefficient of 99.82%, which increases by 1.88% in comparison to the former result obtained by 1st order. In other words, the frequency linearly varies with acceleration within a range of 0 to 1000 g. The sensitivity of the proposed differential resonant graphene accelerometer is confirmed as 21,224 Hz/g over the range, which is remarkably higher than those obtained in previously reported works [19,36].

**Figure 6:**
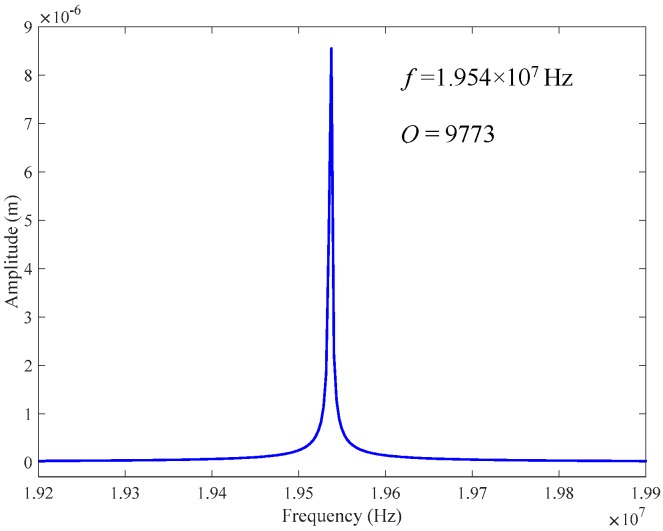
Amplitude–frequency response of the accelerometer at room temperature.

The quality factor is one of the important parameters for resonant sensors, which is strongly related to the energy loss speed in each oscillation cycle. For resonant accelerometer, higher *Q* means stronger ability to resist external interference, better working stability and easier detection of resonance frequency shift. Figure 6 shows the amplitude versus frequency response of the proposed accelerometer at room temperature (293.15 K). The *Q* factor can be calculated by $f_{0}/\Delta f$, where $f_{0}$ is natural frequency and Δf is the bandwidth of the amplitude-frequency curve. The natural frequency $f_{0}$ in Figure 6 is determined as 1.954 × 10^7^ Hz, which is consistent with the simulation result mentioned above. The corresponding calculated *Q* value is 9773, which is by far larger than the previous results obtained at room temperature, such as a *Q* factor of 1000 for the epitaxial graphene resonator in Reference [24] and *Q* factor of 3000 for the drum graphene resonator in Reference [25]. It is also larger than the graphene resonator fabricated by using the resist shrinkage-induced strain (*Q* *=* 7000) in identical dimension order (μm) [14]. In this study, the increase of prestress and non-proof mass adhered on the graphene are regarded as the main reasons of quality factor’s improvement. Due to the ultra-thin structure of graphene beam, when a larger strain is imposed upon the graphene beam, the type of vibration will change from flexural to tension vibration, therefore leading to the decrease in thermoelastic damping [14]. In addition, other than the studies reported before [19,20,21], since no proof mass is attached to graphene beam, the vibration of this ultraclean graphene will be free from the suppression of attached mass. As a consequence, the resonance frequency could obviously be improved, in addition to the declined energy dissipation during oscillation, which enables higher *Q* factor accordingly [24,37].

To sum up, the simulation results in this study demonstrate the excellent performance of the suggested differential resonant graphene accelerometer, such as ultrahigh sensitivity, high resonance frequency and wide acceleration range, owing to the excellent mechanical properties of graphene with high prestress, non-proof mass sensitive structure and optimized folded support beam design. It is worth noting that, although the simulation analyses in this paper are performed under non-damping and room temperature conditions, the results obtained herein could provide great potential in applications of high-g accelerometers wherein the thermal and damping effects would be considered.

## 5. Conclusions

An ultrahigh-sensitivity, differential resonant graphene accelerometer which can offer high-g acceleration measurement is designed and investigated via COMSOL Multiphysics in this paper. The simulation results agree well with the theoretical resonance frequency in consideration of prestress in graphene beams, thus verifying the applications of the graphene accelerometer. In order to enable both of the two graphene beams in resonant condition, the prestress of graphene beams is set as 3 × 10^9^ N/m^2^ (3 GPa) based on simulation analysis and theoretical model. The resonant response simulation of the designed accelerometer demonstrates an ultrahigh sensitivity of 21,224 Hz/g, which is significantly better than the results reported in previous studies. Furthermore, a superior *Q* factor of 9773 is achieved, which well represents the accelerometer’s excellent stability and anti-interference ability. Therefore, these effective efforts validate the advantages of resonant graphene accelerometer over conventional quartz and silicon devices in high-g measurement and provide potential applications in the field of NEMS inertia measurement.

## Figures and Tables

**Table 1:** Initial structural parameters for the graphene accelerometer.

Description	Length (μm)	Width (μm)	Thickness (or Height) (μm)
Substrate	70	70	6
Convex platform	24	14	7
Sensitive mass plate	60	60	5
Sensitive mass block	53	53	5
Folded support beams	46	0.2	5
Graphene resonant beams	10	1	0.335 × 10^−3^
